# Human induced pluripotent stem cell-derived cardiomyocytes for disease modeling and drug discovery

**DOI:** 10.3389/fbioe.2025.1687840

**Published:** 2025-10-16

**Authors:** Taijun Moriwaki, Hidenori Tani, Shugo Tohyama

**Affiliations:** ^1^ Department of Clinical Regenerative Medicine, Fujita Medical Innovation Center, Fujita Health University, Ohta, Tokyo, Japan; ^2^ Kanagawa Institute of Industrial Science and Technology (KISTEC), Kawasaki, Kanagawa, Japan; ^3^ Keio University Regenerative Medicine Research Center, Kawasaki, Kanagawa, Japan

**Keywords:** human induced pluripotent stem cells, cardiac organoids, engineered heart tissue, disease modeling, drug discovery

## Abstract

Human induced pluripotent stem cells (hiPSCs) have emerged as a promising platform for elucidating disease mechanisms and developing new drugs. Over the past 2 decades, it has become possible to efficiently generate large quantities of cardiomyocytes (CMs) from hiPSCs, thereby enabling the reproduction of disease-specific characteristics in culture dishes. Although this technology has the potential to substantially enhance the efficiency of drug discovery and understanding of disease, the immaturity of hiPSC-derived CMs (hiPSC-CMs) has been a major barrier to their widespread adoption. This review discusses the recent advances that address these challenges and explores the potential of hiPSCs to advance disease modeling, elucidate disease mechanisms, and accelerate drug discovery.

## 1 Introduction

Cardiovascular disease is reported to cause approximately 19.8 million deaths annually, ranking as the leading cause of death worldwide (Mensah et al., 2023). Recent analysis has shown that the number of new cardiovascular drugs is steadily decreasing (Figtree et al., 2021). This is mainly because only approximately 5% of new molecular entities (NMEs) are ultimately approved, and the approval of a single NME requires considerable time and expense (Schuhmacher et al., 2016). A fundamental reason for this low success rate is the lack of preclinical models that can accurately evaluate therapeutic efficacy and safety in humans (Schuhmacher et al., 2016). Animal models have been widely used in preclinical trials. However, due to differences in cardiac biology between species, these models have limited ability to predict the efficacy of new drugs and harmful cardiovascular side effects in humans (Milani-Nejad and Janssen, 2014). For example, the heart rate of mice is approximately eight times higher than that of humans, and cardiac repolarization in mouse cardiomyocytes (CMs) depends mainly on transient outward K^+^ current I_to_, 4-aminopryridine sensitive K^+^ current I_K, slow1_, TEA-sensitive K^+^ current I_K, slow2_, and steady-state current I_SS_ ion currents (in humans, depends mainly on I_Ks_ and I_Kr_) (Garg et al., 2018). Additionally, human primary CMs rapidly dedifferentiate during cultivation, making it impossible to accurately evaluate the effects of drugs on the heart (Bird et al., 2003). Therefore, models that can accurately evaluate drug efficacy and cardiotoxicity in humans are essential for drug discovery. A deeper understanding of the disease mechanism is essential for drug discovery to target the early stages of disease development and to develop sensitive and effective drugs with fewer side effects (Bekhite and Schulze, 2021). Disease-specific gene-modified mice and rats, as well as patient-derived primary CMs, have been used to elucidate disease mechanisms. However, as mentioned earlier, there are discrepancies between the human heart and animal or human primary cardiac muscle cells, necessitating models that closely resemble the human heart *in vivo*.

Human induced pluripotent stem cells (hiPSCs) have unlimited proliferative potential and can generate patient-derived hiPSC-derived CMs (hiPSC-CMs), making them extremely useful tools for elucidating disease mechanisms and for subsequent drug development. Since the report by Takahashi and Yamanaka et al. on the successful reprogramming of human fibroblasts into hiPSCs, research has been actively conducted on the differentiation of hiPSCs into target cells (Takahashi et al., 2007). In the field of cardiology, protocols for differentiating hiPSCs into CMs have continuously improved, resulting in a substantial increase in the efficiency of differentiation into CMs compared to when hiPSCs first became available (Feeney et al., 2025; Umei et al., 2025). Furthermore, a method for removing non-cardiac cells obtained concomitantly with the differentiation of hiPSCs into CMs and technology for the mass production of hiPSC-CMs have been developed, enabling the simple and large-scale production of high-purity hiPSC-CMs (Someya et al., 2021; Tanosaki et al., 2020; Tohyama et al., 2017; Tohyama et al., 2016; Tohyama et al., 2013). However, hiPSC-CMs exhibit an immature phenotype similar to that of fetal CMs, which limits their application in elucidating disease mechanisms and drug discovery (Kannan and Kwon, 2020; Tu et al., 2018). Cardiovascular diseases are common in the elderly; therefore, the maturation of hiPSC-CMs is key to producing cardiovascular disease models that are useful for drug discovery.

This review provides an overview of the differences between hiPSC-CMs and adult human CMs (AdCMs) and considers various studies aimed at achieving cardiomyocyte maturation to more accurately reproduce the phenotype of AdCMs. Furthermore, we introduce research on disease and drug induced cardiotoxicity modeling and drug discovery using hiPSC-CMs that utilize the maturation technologies developed to date.

## 2 Differences between hiPSC-CMs and AdCMs

**FIGURE 1:**
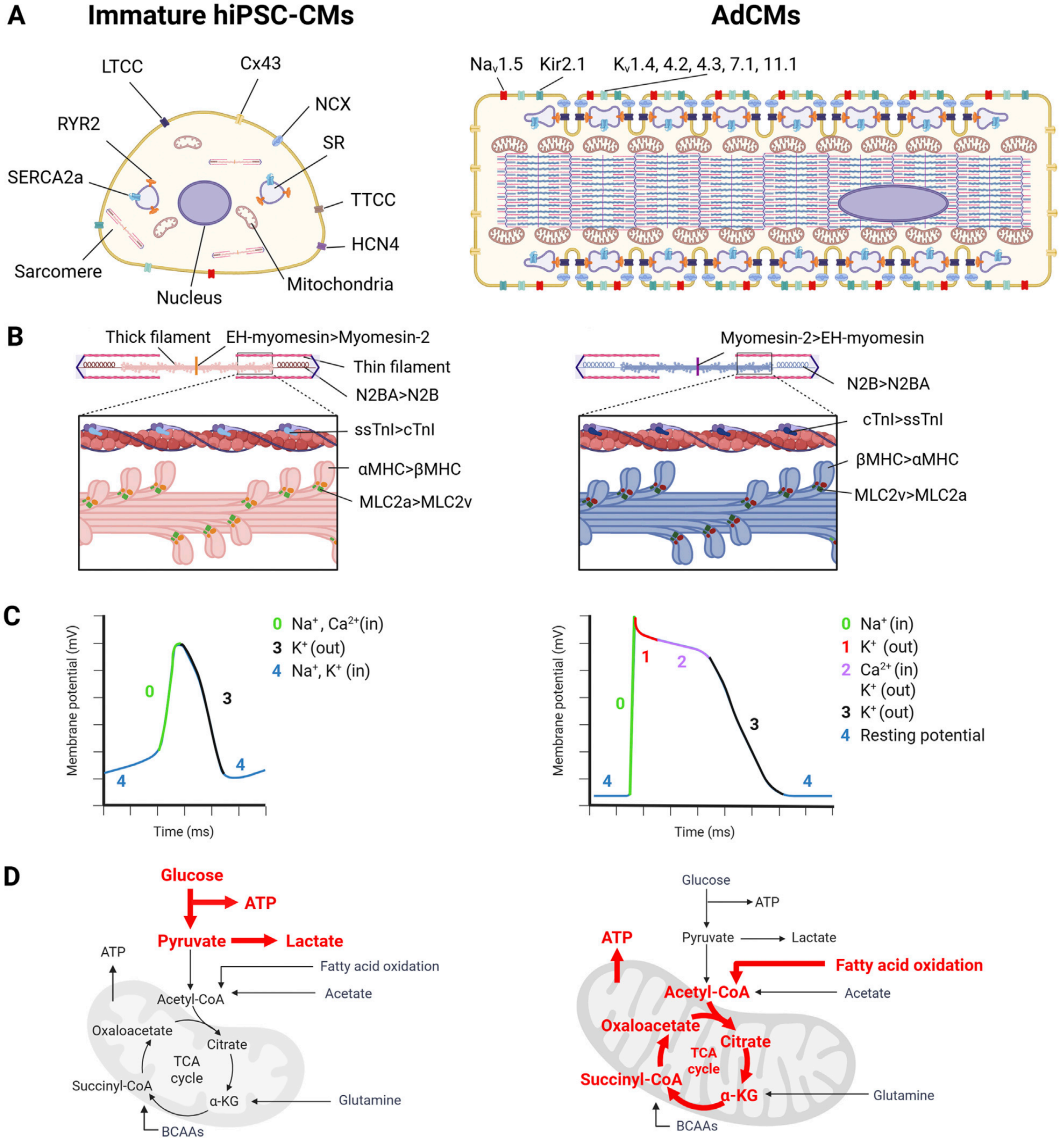
The difference between human induced pluripotent stem cell-derived cardiomyocytes (hiPSC-CMs) and adult human cardiomyocytes (AdCMs) in **(A)** cardiomyocyte (CM) structure, **(B)** sarcomere isoform, **(C)** electrophysiology, **(D)** metabolism. This image was created in BioRender. Fujita, T. (2025) https://BioRender.com/zr1cw9z.

The hiPSCs have the potential to accurately reproduce the genetic and phenotypic characteristics of cardiovascular diseases *in vitro*, making them a highly attractive tool for developing disease models. However, hiPSC-CMs are immature compared to AdCMs, which limits their application in elucidating disease mechanisms and drug discovery. This review focuses on cell morphology, electrophysiology and metabolic maturation (Figures 1A–D).

### 2.1 Cell morphology

AdCMs have a cylindrical shape with a volume of approximately 40,000 μm^3^ (140 µm in length, 20 µm in width) (Smith and Bishop, 1985; Gerdes et al., 1992). In contrast, hiPSC-CMs are smaller than AdCMs, with a volume of 3000–6000 μm^3^ and a more rounded shape (Rupert et al., 2017; Josvai et al., 2025).

The sarcomere is the functional unit responsible for cell contraction. Immature hiPSC-CMs have poorly-organized sarcomeres, which are randomly orientated, whereas AdCMs form myofibrils parallel to the entire CMs (Figure 1A) (Bedada et al., 2016; Ahmed et al., 2020). The sarcomere consists of thin and thick filaments and Z-bands (Figure 1B). The sarcomere length of hiPSC-CMs is 1.7–2.0 μm, which is slightly shorter than that of AdCMs (1.9–2.2 μm). Various proteins that constitute the sarcomere undergo isoform switching as maturation progresses (Figure 1B). For example, αMHC is expressed in immature CMs and βMHC is expressed in mature CMs in humans, whereas βMHC is expressed in the fetal stage and αMHC is expressed in the adult stage in rats and mice (Lompre et al., 1979; Lompré et al., 1984). The regulatory light chain of myosin is predominantly MLC2a in immature CMs, and switches to MLC2v as maturation progresses (O'Brien et al., 1993; Kubalak et al., 1994). Slow-twitch skeletal troponin I (ssTnI) is expressed in immature CMs and switches to the expression of cardiac TnI (Gorza et al., 1993). For titin, the immature CMs express the long and flexible titin isoform, N2BA, whereas mature CMs express the short and rigid titin isoform, N2B (Lahmers et al., 2004). Myomesin isoforms that constitute the M-band express EH-myomesin (gene name: *MYOM1*) in immature CMs and switch to Myomesin-2, which lacks the EH domain, in mature CMs (Agarkova et al., 2000). In heart failure (HF) CMs, sarcomere isoform switching also occurs. HF causes isoform switching opposite to maturation (i.e., switching from N2B to N2BA of titin, from Myomesin-2 to EH-myomesin, and from βMHC to αMHC) (Miyata et al., 2000; Krüger and Linke, 2009; Schoenauer et al., 2011).

T-tubules are structures observed during the late stage of myocardial maturation in the mouse heart after birth (P10) (Xu et al., 2024). Therefore, they are rarely observed in hiPSC-CMs, leading to delayed calcium-induced calcium release (CICR) due to the spatial uncoupling between L-type Ca^2+^ channels (LTCC) and RYR2 (Lieu et al., 2009). The biogenesis and maintenance of T-tubules involve various membrane scaffolding proteins, such as bridging integrator 1 (BIN1), junctophilin 2 (Jph2), and caveolin 3 (Cav3) (Hong et al., 2014; Zhang et al., 2014; Bryant et al., 2018). In particular, BIN1 has recently been shown to regulate T-tubule proliferation and organization in collaboration with MTM1 and DNM2 (Perdreau-Dahl et al., 2023). Additionally, recent studies have shown that Ptpn23 plays an adapter role between the dystrophin glycoprotein complex and the Z-band of the sarcomere and is essential for T-tubule biogenesis and maintenance (Xu et al., 2024). Regular T-tubules are formed in AdCMs, whereas they are barely formed in immature hiPSC-CMs. Therefore, the underdevelopment of T-tubules leads to the disassociation of LTCC and RYR2, resulting in delayed CICR. The expression of BIN1 and Cav3, which are involved in T-tubule formation, increases with hiPSC-CM maturation (Soma et al., 2024). In HF CMs, T-tubule formation is disrupted, or T-tubule density is reduced (Bryant et al., 2018; Xu et al., 2024).

Morphological differences were also observed in mitochondria. AdCMs mitochondria are large with well-developed cristae (Li et al., 2020a). The development of the cristae increases the surface area of the inner membrane and promotes efficient cellular respiration. In contrast, hiPSC-CM mitochondria are smaller than those of AdCMs and have absent or sparse cristae (Wang et al., 2023b).

### 2.2 Electrophysiology

The action potential of CMs is formed by Na^+^ current I_Na_ via Na_v_1.5 (phase 0); I_Kto_ (phase 1); L-type and T-type Ca^2+^ currents I_Ca_, which contribute to the plateau phase (phase 2); I_kr_, I_Ks_ (phases 2 and 3); and the inwardly rectifying K^+^ current I_K1_ and pacemaker current I_f_ (phase 4) (Karakikes et al., 2015). Immature hiPSC-CMs differ from AdCMs in five aspects (Figure 1C). First, hiPSC-CMs express potassium hyperpolarization-activated cyclic nucleotide-gated channel 4 (HCN4), which generates inward currents at the diastolic potential, enabling autonomous beating (Li et al., 2020b). In contrast, AdCMs express low levels of HCN4, resulting in the absence of autonomous beating (Saito et al., 2015). Second, hiPSC-CMs have insufficient expression of Kir2.1 (*KCNJ2*) and Kir2.2 (*KCNJ12*), resulting in a smaller I_K1_ and a higher resting membrane potential (approximately −60 mV) compared to AdCMs (approximately −90 mV) (Goversen et al., 2018). Third, hiPSC-CMs have lower Na_v_1.5 (*SCN5A*) expression than AdCMs, resulting in a 50% slower rise in upstroke velocity (Lee et al., 2024b). Fourth, hiPSC-CMs express lower levels of Cav1.2 (*CACNA1C*) than AdCMs and lead to a lack or shorter plateau phase (Yang et al., 2014a). Finally, the conduction velocity of hiPSC-CMs (0.03–0.06 m/s) is slower than that of AdCMs (0.3–0.4 m/s) (Durrer et al., 1970; Bursac et al., 1999; Kadota et al., 2013; Zhang et al., 2013). This is because immature hiPSC-CMs have a lower expression of connexin 43 than AdCMs, and connexin 43 is distributed around the cells, whereas AdCMs have connexin 43 localized in the intercalated disk (Gilbert, 2021). As described above, there are several electrophysiological differences between hiPSC-CMs and AdCMs. When evaluating hiPSC-CMs for applications such as assessing the proarrhythmic effects of new drugs, it is important to note that immature hiPSC-CMs only partially reflect the electrophysiology of AdCMs.

Ca^2+^ that flows into the cell through LTCC activates RYR2, releasing large amounts of Ca^2+^ from the sarcoplasmic reticulum (SR) into the cytoplasm (Endo, 1977). This process is known as CICR. The presence of the T-tube brings RYR2 and LTCC in close proximity, enabling efficient calcium processing (Bers, 2002). CICR leads to a rapid increase in Ca^2+^ concentration; Ca^2+^ binds to cTnC, initiating sarcomere contraction (Parmacek and Solaro, 2004). In contrast, during the diastolic phase, Ca^2+^ in the cytoplasm is taken up into the SR via SERCA2a (gene name: *ATP2A2*) and simultaneously released outside the cell via NCX (Aronsen et al., 2016). Of these proteins, NCX expression is unchanged between hiPSC-CMs and AdCMs, whereas RYR2, SERCA2, and Cavβ are higher in AdCMs than in hiPSC-CMs (Rao et al., 2013).

### 2.3 Metabolism

CM metabolism undergoes dramatic changes during fetal and adult development. During the fetal stage, cardiac muscle cells obtain ATP mainly through glycolysis due to the low-oxygen environment, mitochondrial immaturity, low levels of fatty acids, and high levels of lactate in the blood (Lopaschuk and Jaswal, 2010; Ding et al., 2021; Chen et al., 2023). The hiPSC-CMs also depend on glycolysis for ATP production (Figure 1D) (Kim et al., 2013). In AdCMs, oxidative phosphorylation (OXPHOS) accounts for almost all ATP demand (about 95%), and the majority of mitochondrial ATP production is derived from the oxidation of fatty acids (Lopaschuk et al., 2021). This leads to an increase in mitochondrial size, elongation, and membrane potential as well as the acquisition of more developed cristae during CM maturation (Scuderi and Butcher, 2017). Healthy AdCMs require large amounts of ATP to maintain their contractile function and they rely on OXPHOS, which is more efficient for ATP production than glycolysis. In contrast, in end-stage HF, the ATP content decreases by up to 30% compared to that in healthy hearts due to reduced mitochondrial oxidative ability (Ingwall and Weiss, 2004; Bottomley et al., 2013). In HF cardiomyocytes, glycolysis is increased to compensate for reduced ATP production (Allard et al., 1994).

## 3 Various approaches for enhancing hiPSC-CM maturation

**FIGURE 2:**
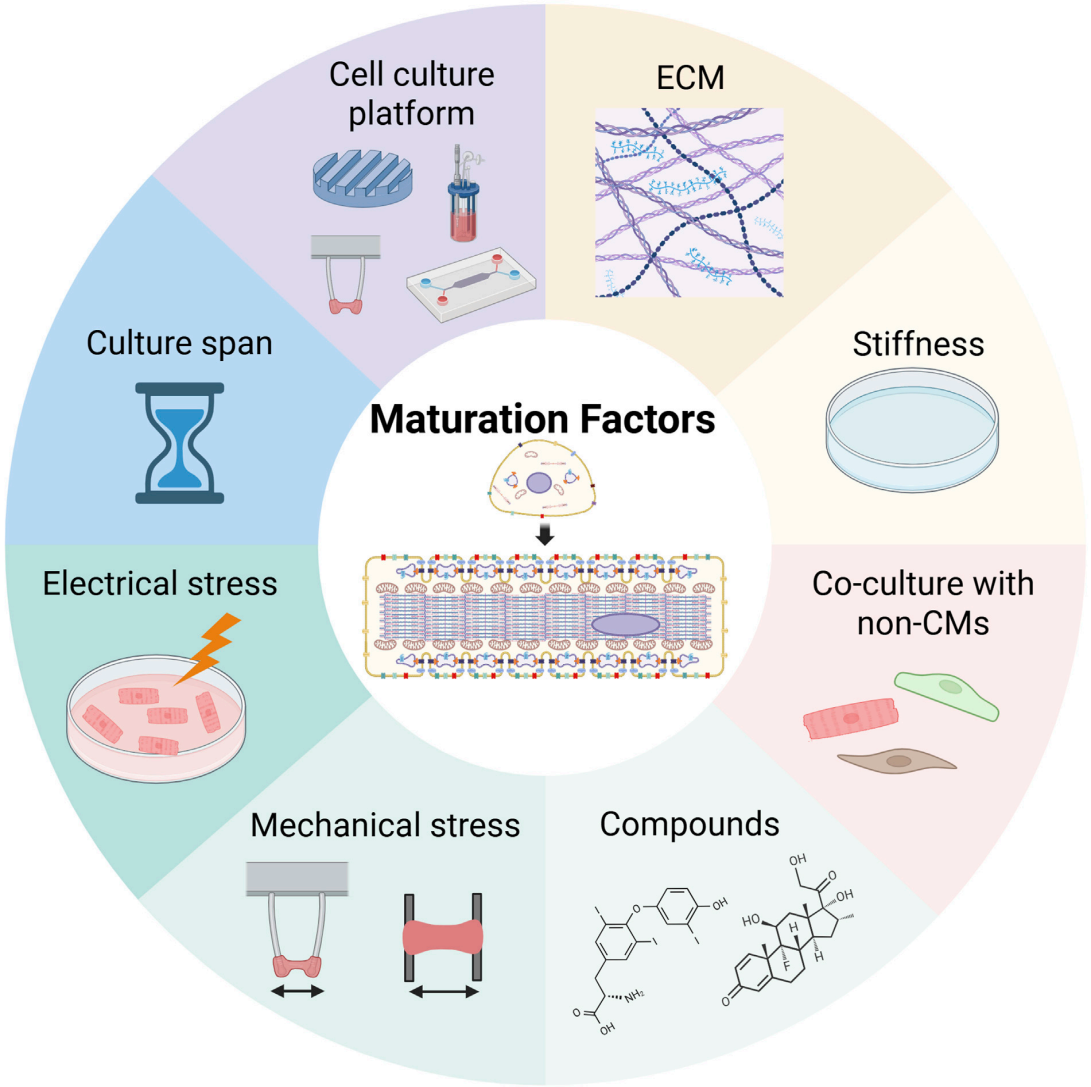
Maturation factors of human induced pluripotent stem cell-derived cardiomyocytes. This image was created in BioRender. Fujita, T. (2025) https://BioRender.com/zr1cw9z.

Generally, hiPSC-CMs are more similar to fetal CMs than to AdCMs. Since many cases of HF occur in the elderly, it is important to enhance hiPSC-CM maturation to produce HF models. The factors that regulate the maturation of hiPSC-CMs are listed below (extracellular matrix, ECM; culture substrate stiffness; co-culture with non-CMs; biological and chemical compounds; electrical and mechanical stimuli; culture span; and cell culture platforms) (Figure 2). A combination of these factors is expected to enhance the maturation of hiPSC-CMs.

### 3.1 ECM

The ECM regulates cellular behavior, such as cell migration, proliferation, and differentiation during heart development, and provides structural support (Song and Zhang, 2020; Silva et al., 2021b; Thomas et al., 2022). Barreto-Gamarra focused on the increased expression of α2β1 integrin in cardiac progenitor cells (CPCs) and demonstrated that using Type I collagen as the ECM at the hiPSC-CPC stage enhanced the maturation of hiPSC-CMs (Barreto-Gamarra and Domenech, 2025). Chanthra et al. screened ECM components that contributed to hiPSC-CM maturation and demonstrated that laminin 511/521 promoted hiPSC-CM maturation (Chanthra et al., 2020). We extracted porcine collagen from organs, such as the heart, kidney, lung, liver, spleen, and skin, and investigated which organ-derived collagen was suitable for hiPSC-CM-derived heart tissue maturation. Collagen from the heart exhibited the highest degree of hiPSC-CM maturation and shape retention rate in heart tissue, indicating that Type III and Type V collagen within the heart play important roles (Tani et al., 2023). These results indicate that the ECM contributes to hiPSC-CM maturation, suggesting the necessity of selecting an appropriate ECM for mature hiPSC-CMs.

### 3.2 Culture substrate stiffness

The elastic moduli of the neonatal and adult hearts are approximately 6.8 kPa and 25.6 kPa, respectively (Bhana et al., 2010). The elastic moduli of plastic and glass dishes commonly used for cell culture are >1 GPa and >70 GPa, respectively, and hiPSC-CMs cultured in these dishes are not in an environment with appropriate stiffness (Travers et al., 2016). To create an environment similar to *in vivo* conditions, polyacrylamide or polydimethylsiloxane was used as a culture substrate (Pasqualini et al., 2015; Herron et al., 2016; Körner et al., 2021). Various maturation indicators such as increased cell size, sarcomere length, connexin 43 expression, conduction velocity, and sarcomere maturation have been reported to be higher in hiPSC-CMs seeded on conventional culture substrates (Pasqualini et al., 2015; Herron et al., 2016; Körner et al., 2021; Dhahri et al., 2022).

### 3.3 Co-culture with non-CMs

The heart is composed of various cells, including CMs, vascular endothelial cells, and cardiac fibroblasts, with CMs accounting for only 25%–35% of the total number of cells (Nag, 1980; Bergmann et al., 2015; Pinto et al., 2016). In adult mouse hearts, vascular endothelial cells (approximately 60%) and cardiac fibroblasts (approximately 15%) constitute the largest proportion of non-CMs (Pinto et al., 2016). Because these non-CMs contribute to the production of ECMs, the supply of various signaling pathways and growth factors in place of CMs, co-culture of these cells with hiPSC-CMs have been shown to improve their electrophysiological maturation, sarcomere alignment, and metabolic function (Yang et al., 2023).

### 3.4 Biological and chemical compounds

Thyroid and glucocorticoid hormones are essential for heart maturation (Li et al., 2014; Rog-Zielinska et al., 2015). The administration of the thyroid hormone, T3, to hiPSC-CMs has been shown to contribute to a wide range of hiPSC-CM maturation processes, including increased cell size, sarcomere length, contractile force, and mitochondrial maturation (Lee et al., 2010; Chattergoon et al., 2012; Yang et al., 2014b). The combination of T3 and glucocorticoids enhances T-tube development, CICR, and ventricular-like excitation–contraction coupling (Parikh et al., 2017). NRG1, a member of the epidermal growth factor (EGF) family, is essential for cardiac conduction system development, and its administration to hiPSC-CMs has been shown to promote the maturation of metabolism and contractility (Rupert and Coulombe, 2017). Estrogen-related receptor γ (ERRγ) agonists and S-phase kinase-related protein inhibitors (S-PK2) produce marked upregulation in TNNI3 expression (Miki et al., 2021). In particular, hiPSC-CMs treated with the ERRγ agonist produce a larger cell size, longer sarcomere length, the presence of T-tubules, and have enhanced metabolic function and contractile and electrical properties.

Other studies have focused on the metabolic transition from glycolysis to OXPHOS and fatty acid oxidation (FAO) during the maturation of CMs (Horikoshi et al., 2019; Feyen et al., 2020). Feyen et al. reported that hiPSC-CMs cultured in media containing low levels of glucose and high levels of fatty acids exhibited increased mitochondrial numbers with more aligned Z-lines, increased expression of mature CM-related genes, increased contractility, and electrophysiological maturation. This maturation medium made it possible to reliably model LQT3 and dilated cardiomyopathy (Feyen et al., 2020). Peroxisome proliferator-activated receptors (PPARs) are ligand-activated transcription factors involved in growth, proliferation, and metabolism, with three isoforms: PPARα, PPARδ, and PPARγ (Barger and Kelly, 2000; Ahmadian et al., 2013). Activation of PPARα and PPARδ has been shown to contribute to the maturation of hiPSC-CMs (Wickramasinghe et al., 2022; Lee et al., 2024a). Activation of PPARδ increases the content of mitochondria and peroxisomes, enhances cristae formation, and increases FAO flux, thereby inducing a metabolic switch from glycolysis to FAO (Wickramasinghe et al., 2022).

### 3.5 Electrical and mechanical stimulation

Since the membrane potential and contractility of CMs change with cardiac growth and development, electrical and mechanical stimuli play crucial roles in the maturation of hiPSC-CMs. Tan et al. demonstrated that the incorporation of electrically-conductive silicon nanowires into hiPSC-CM spheroids improved the intrinsic electrical microenvironment, thereby promoting the structural and functional maturation of hiPSC-CMs (Tan et al., 2015). The combination of nanowires and electrical stimulation enhanced cell–cell junction formation, improved the development of contractile machinery, and led to a marked decrease in the spontaneous beat rate of hiPSC-CM spheroids (Richards et al., 2016). Exogenous electrical pulses similar to those in CMs promote the differentiation and functional maturation of iPSC-CMs (Ma et al., 2016).

As for mechanical stimulation, passive stretching, guided by computational modeling, regulates the alignment and calcium dynamics of hiPSC-CMs in engineered heart muscle (EHM) (Abilez et al., 2018). LaBarge et al. evaluated the maturation of scaffold-free hiPSC-CM spheroids exposed to electrical stimulation with an electric field of 6.5 V/cm with 5 ms pulses at a frequency of 2 Hz for 7 days and mechanical stimulation at 10% strain at a frequency of 1 Hz for 7 days. The hiPSC-CMs increased the expression of gap junctions and calcium-handling mechanisms at the transcriptional, protein, and ultrastructural levels (LaBarge et al., 2019). Although this study did not directly compare the maturation of hiPSC-CMs between electrical and mechanical stimulation, the data are useful because the authors evaluated the maturation of hiPSC-CMs exposed to both electrical and mechanical stimulation using the same assessment. Electrical and mechanical stimulation thus contribute to the maturation of hiPSC-CMs.

### 3.6 Culture span

In humans, a long time is required for the fetus to mature into an adult. Therefore, long-term culture is one approach to the maturation of hiPSC-CMs. In initial studies, CMs differentiated from human embryonic stem cells (hESCs) were cultured for 60 days and exhibited more mature characteristics, such as increased cell size, reduced cell proliferation, and organized sarcomeres (Snir et al., 2003). The hiPSC-CMs cultured for approximately 1 year (Day 360) produced myofibrils that were more densely packed and appeared to have mature Z, A, H, and I bands after 180 days of long-term culture (Kamakura et al., 2013). The appearance of M-bands was observed only after 360 days of long-term culture. The hiPSC-CMs can mature over long-term culture but this process is time-consuming and costly.

### 3.7 Cell culture platforms

#### 3.7.1 Two-dimensional (2D) platforms

The hiPSC-CMs cultured in 2D typically exhibit limited maturity. Cell micropatterning has the potential to enhance the maturation of hiPSC-CMs in 2D platforms. Mature cardiomyocytes are rod-shaped; patterning the culture substrate so that cells adhere in this orientation aligns the sarcomeres and leads to electrophysiological maturation (Al Sayed et al., 2024). Combining micropatterning with substrates of appropriate stiffness has been shown to enhance the maturity of hiPSC-CMs (Tsan et al., 2021; Strimaityte et al., 2022). This regulation of micropatterning and substrate stiffness enables T-tubule formation, which is difficult to achieve in 2D culture (Strimaityte et al., 2022). Cell density also influences the maturation of hiPSC-CMs. Increasing the cell density upregulates the expression of Kir2.1 (*KCNJ2*), enhancing electrophysiological maturation (Li et al., 2020c). Recent advances have enabled the production of highly-mature hiPSC-CMs in 2D culture by combining various maturation methods. However, as 2D platforms do not accurately reproduce the complex structure and dimensionality of the original tissue, their ability to mimic specific aspects of development, physiological functions, and diseases is limited.

#### 3.7.2 Three-dimensional (3D) platform

Three-dimensional (3D) platforms mimic the *in vivo* environment more closely than 2D platforms, and many research groups have reported the advantages of culturing hiPSC-CMs in 3D systems to enhance their maturity (Jha et al., 2016; Tiburcy et al., 2017; Ronaldson-Bouchard et al., 2018; Ulmer et al., 2018; Beauchamp et al., 2020). The 3D tissues can be divided into scaffold-free and scaffold-based systems (Tani and Tohyama, 2022).

Scaffold-free 3D tissues include cardiac spheroids containing only hiPSC-CMs; cardiac microtissues created by mixing hiPSC-CMs with other cell types, such as endothelial cells and cardiac fibroblasts; and cardiac organoids developed from embryoid bodies (EBs) (Richards et al., 2017; Hofbauer et al., 2021; Lewis-Israeli et al., 2021; Silva et al., 2021a; Lee et al., 2022; Moriwaki et al., 2023; Schmidt et al., 2023). The formation of hiPSC-CM spheroids has been shown to results in greater structural, metabolic, and functional maturation than hiPSC-CMs cultured in a 2D platform (Jha et al., 2016; Correia et al., 2018). Co-culture with non-cardiac cells such as cardiac fibroblasts has been shown to enhance maturation (Kahn-Krell et al., 2022). Recent advances in the manufacturing technology of homogeneous spheroids and microtissues have been shown to improve the reproducibility of drug responses and are expected to contribute to drug discovery and the elucidation of disease mechanisms (Chen et al., 2019; Moriwaki et al., 2023; Moriwaki et al., 2025). Cardiac organoids are characterized by being derived from EBs and mimic cardiac development. They possess chamber cavities, an endocardium, and an epicardium, making them more similar to the heart in terms of structure, cell type, and spatial arrangement (Hofbauer et al., 2021; Lewis-Israeli et al., 2021). Schmidt et al. successfully differentiated the first heart field, anterior second heart field (aSHF), and posterior SHF from hiPSCs, producing left ventricular, right ventricular, and atrial organoids with chamber cavities, respectively (Schmidt et al., 2023). Organoids resembling the early stages of heart development were produced by combining these organoids, in which electrical signals flowed from the atrial organoid to the left and right ventricular organoids. Although discrepancies in size and cell composition ratio exist in comparison to cardiac spheroids and cardiac microtissues, these cardiac organoids are an attractive platform for evaluating fetal cardiovascular diseases and the effects of teratogens on early heart development due to their ability to mimic heart development.

Scaffold-based 3D tissues include engineered heart/cardiac tissues (EHT/ECT), biowires, and heart-on-a-chip (HoC). These tissues use collagen, fibrin, and decellularized matrices as cellular scaffolding materials and play a crucial role in the formation of *in vivo*-like structures and the dynamic contractile properties of cardiac tissues (Zimmermann et al., 2002; Hansen et al., 2010; Blazeski et al., 2019; Goldfracht et al., 2019). Of these approaches, EHT is widely used for disease modeling and drug testing and has shown promising results in therapeutic applications (Lemoine et al., 2018; Mannhardt et al., 2020). EHT is a cardiac tissue composed of cardiomyocytes and interstitial cells embedded in an environment supported by two flexible pillars with fibrin, collagen, or a decellularized matrix as the scaffold. The pillars supporting both ends of the tissue provide diastolic tension and promote cardiomyocyte alignment. Ronaldson-Bouchard et al. successfully produced EHT with a considerably advanced degree of maturity similar to AdCMs (including highly-organized ultrastructural features, physiological sarcomere length (2.2 μm), high mitochondrial density (30%), T-tubule presence, and metabolic maturity) by applying electrical stimulation-based “intensity training” to EHT (Ronaldson-Bouchard et al., 2018). This “intensity training” method involved increasing electrical stimulation by 0.33 Hz per day over a 2-w period, starting at 2 Hz and ending at 6 Hz.

## 4 Comparison of maturation factors

This chapter discusses which of the approaches introduced in Chapter 3 contributes most to hiPSC-CM maturation. The current, most effective strategy for promoting hiPSC-CM maturation is considered to be electrical and mechanical stimulation training using EHT (Ronaldson-Bouchard et al., 2018; Zhao et al., 2019; Lu et al., 2021). Crucially, this electrical and mechanical stimulation training begins in the early stages of hiPSC-CM culture, with stimulation intensity gradually increased each day. Although some electrophysiological parameters remain below AdCM maturation levels (resting membrane potential, −70 mV; action potential duration, approximately 500 ms; upstroke velocity, approximately 25 V/s), the hiPSC-CMs exhibited AdCM-like phenotypes, including a characteristic notch in ventricular action potentials, high conduction velocity (25 cm/s), high contractility (44 mN/mm^2^), sarcomere lengths of 2.2 μm, T-tubule formation, and high mitochondrial density (Ronaldson-Bouchard et al., 2018).

Recently, Li et al. by combining electrical stimulation, micropatterning, and fatty acid-containing media, demonstrated that maturation factors contributed substantially to hiPSC-CM maturation by combining electrical stimulation, micropatterning, and fatty acid-containing medium (Li et al., 2025). Although micropatterning limited maturation primarily to sarcomere organization, electrical stimulation was a key maturation factor for mitochondrial development and metabolic/electrophysiological maturation. This combination achieved a level of maturation comparable to the above-mentioned 3D model in terms of electrophysiological maturation (including a characteristic notch in the action potential, a resting membrane potential of −65.6 mV, and a conduction velocity of 27.8 cm/s).

As described in Chapter 2, the existence of numerous indicators of hiPSC-CM maturation complicates the discussion of which maturation method most comprehensively promotes hiPSC-CM maturation. Therefore, it is desirable to develop indicators to comprehensively evaluate the maturity of hiPSC-CMs.

## 5 Disease models *in vitro*

Numerous models of heart disease have been reported using hiPSC-CMs, including dilated cardiomyopathy (DCM); hypertrophic cardiomyopathy (HCM); cardiac channelopathies, such as long QT syndrome (LQTS) and Brugada syndrome (BrS); and myocardial infarction (MI)/ischemic reperfusion (IR) injury (Ewoldt et al., 2025). The culture platforms and maturation approaches used to generate the disease models are listed in Table 1. Channelopathies are limited to 2D culture platforms, and most hiPSC-CM maturation methods use long-term culturing (El-Battrawy et al., 2019; Chang et al., 2021; Sun et al., 2023). Because these channelopathies cause abnormalities in action potentials, these models always utilize either a patch clamp, a multi-electrode assay, or both, to evaluate action potentials. In contrast, DCM and HCM models predominantly use EHT as their culture platform (Fomin et al., 2021; Huang et al., 2023; Ewoldt et al., 2024). These maturation approaches use various combinations of co-culture, culture duration, and electrical stimulation. Since quantifying contractility is crucial for evaluating these disease models, hiPSC-CM movement was assessed using video recording. Particularly with EHT, the contractile force can be estimated by tracing the movement of the EHT post (Seeger et al., 2019). MI/IR injury models use various culture platforms, including 2D monolayers, HoC, and cardiac organoids (Gaballah et al., 2022; Veldhuizen et al., 2022). These models frequently assess cell survival rates or calcium transients using Cal520 to evaluate arrhythmias.

**TABLE 1:** hiPSC-CMs based disease model and drug induced cardiotoxicity model.

Disease model	Authors	Culture platform	Maturation factors
TTNtv-DCM	Hinson et al. (2015)	Fibrin/Collagen I-based EHT	Coculture: hiPSC-CMs, hMSCs (93:7)
	Fomin et al. (2021)	Collagen I/Matrigel-based EHT	Coculture: hiPSC-CMs, hFFs (7:3) Culture span: 4 weeks (From the formation of the EHT)
	Huang et al. (2023)	Collagen I/Matrigel-based EHT	Coculture: hiPSC-CMs, hCFs (9:1)
LMNA-DCM	J.Lee et al., 2019	2D monolayer	―
	Shah et al. (2021)	3D micropatterned cardiac cultures	―
	Qiu et al. (2024)	Fibrin/Matrigel-based EHT	―
HCM	Seeger et al. (2019)	Fibrin/Matrigel-based EHT	Culture span: Day 35–50
	Wu et al. (2019)	2D monolayer	Culture span: Day 30
	Ewoldt et al. (2024)	Fibrin/Matrigel-based EHT	Coculture: hiPSC-CMs, vCFs (9:1)
	Lan et al. (2013)	2D monolayer	―
	Wang et al. (2023a)	Collagen I/Matrigel/Fibrin-based EHT	Culture span: hiPSC-CMs, vCFs (10:1) Electrical stimulation: 1∼5.2 Hz Culture span: 7∼10 weeks (From the formation of the EHT)
LQT1	Moretti et al. (2010)	2D monolayer	―
	Takaki et al. (2019)	2D monolayer	―
LQT2	Matsa et al. (2011)	2D monolayer	Culture span: Day 25∼30
	Mesquita et al. (2019)	2D monolayer	Culture span: Day 30∼45
	Chang et al. (2021)	2D monolayer	Culture span: Day 60
LQT3	McKeithan et al. (2020)	2D monolayer	Maturation medium^a^
BrS	Sun et al. (2023)	2D monolayer	Culture span: Day 30∼40
	El-Battrawy et al. (2019)	2D monolayer	Culture span: Day 40∼60
	Chavali et al. (2019)	2D monolayer	Culture span: Day 40
SQT1	El-Battrawy et al. (2018)	2D monolayer	Culture span: Day 40∼50
MI/IR injury	Gaballah et al. (2022)	2D monolayer	Culture span: Day 30∼60
	Hidalgo et al. (2018)	2D monolayer	Compounds:10 mM of Galactose, 50 μM of Palmitic acid, and 100 μM of Oleic acid
	Veldhuizen et al. (2022)	3D cardiac tissue on a-chip	Coculture: hiPSC-CMs, hCFs (4:1) Culture span: Day 36
	Song et al., 2024	COs	Culture span: Day 30
	Ellis et al. (2022)	HoC	Coculture: hiPSC-CMs, hiPSC-ECs (5:1)
Cardiotoxicity	Altrocchi et al. (2023)	2D monolayer	―
	Jang et al. (2024)	COs	―
	Liu et al. (2024a)	Fibrin-based EHT	―
		Micropatterning	Culture substrate stiffness: 10 kPa polyacrylamide hydrogel

hMSC: human mesenchymal stem cells, hFFs: human foreskin fibroblasts, hCFs: human cardiac fibroblasts, vCFs: ventricular cardiac fibroblasts, COs: cardiac organoids.

^a^ DMEM without glucose supplemented with 3 mM glucose, DMEM without glucose supplemented with 3 mM glucose, 10 mM L-lactate, 5 μg/mL Vitamin B12, 0.82 μM biotin, 5 mM creatine monohydrate, 2 mM taurine, 2 mM L-carnitine, 0.5 mM ascorbic acid, 1x NEAA, 0.5% (w/v) Albumax, 1 × B27% and 1% KOSR.

**FIGURE 3:**
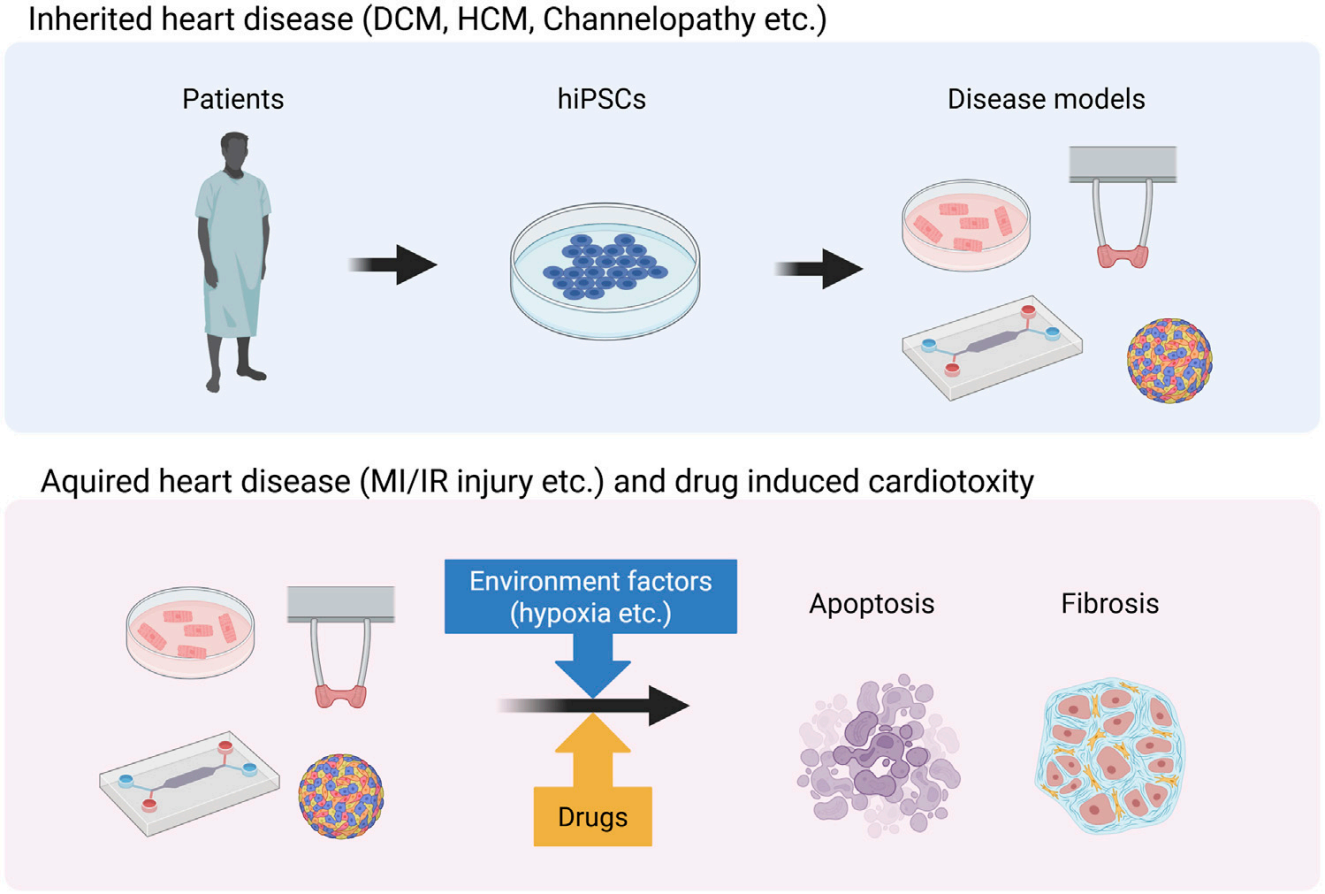
Generation of disease models for inherited and acquired heart disease. This image was created in BioRender. Fujita, T. (2025) https://BioRender.com/zr1cw9z.

When generating models for inherited heart diseases such as DCM, HCM, and channelopathies, patient-derived iPSCs must be established (Figure 3) (Huang et al., 2023; Qiu et al., 2024). Furthermore, the current gold standard involves generating control hiPSCs by repairing the patient’s genetic mutation using genome-editing tools such as CRISPR-Cas9. Non-hereditary heart disease models such as MI/IR injury models are generated by exposing hiPSC-CMs to environmental factors specific to non-hereditary heart diseases (Figure 3) (Ellis et al., 2022). In the MI models, these factors are typically involved in hypoxia.

### 5.1 Dilated cardiomyopathy

Dilated cardiomyopathy (DCM) is a nonischemic heart disease characterized by structural and functional abnormalities of the myocardium. DCM is the most common nonischemic cause of HF (Gigli et al., 2025). The most common genetic mutation in genetic DCM is titin truncating variants (*TTN*tvs), which accounts for approximately 25% of familial cases (Roberts et al., 2015). Early studies using *TTN*tvs hiPSC-CMs demonstrated that titin mutations disrupt the critical link between sarcomere formation and adaptive remodeling (Hinson et al., 2015). Recent studies using hiPSC-CMs have identified two mechanisms underlying the pathophysiology of DCM: the accumulation of *TTN*tvs protein aggregates leads to toxic peptide effects due to abnormalities in the protein quality control (PQC) system and sarcomere defects caused by haploinsufficiency (Fomin et al., 2021; Huang et al., 2023). However, these two studies showed discrepancies in the relative contributions of proteasome- and autophagy-mediated degradation of *TTN*tvs aggregates, suggesting that the selectivity of these two PQC pathways may vary between different cell lines. Additionally, no arrhythmic events were reported in these studies, suggesting that hiPSC-CMs were immature.

*LMNA* mutations are also one of the most common genetic causes of DCM, accounting for 4%–8% of all cardiomyopathies (Schultheiss et al., 2019). The *LMNA* gene encodes a protein that is a major component of the nuclear lamina and plays a crucial role in nuclear and cytoskeletal organization, mechanical stability, chromatin organization, signal transduction, gene regulation, genomic stability, and cell differentiation (Capell and Collins, 2006). Patients with *LMNA*-DCM exhibit prominent early onset life-threatening cardiac electrical abnormalities, such as atrioventricular blocks, ventricular tachycardia, and fibrillation. A study focusing on arrhythmia phenotypes and their associated pathways discovered that the platelet-derived growth factor (PDGF) signaling pathway was activated in *LMNA*-DCM-derived iPSC-CMs and that pharmacological and molecular-level inhibition of the PDGF signaling pathway suppressed arrhythmia phenotypes (Lee et al., 2019). A lamina–chromatin interaction study suggests that the lamina network safeguards cellular identity and that *LMNA* mutations may cause abnormal gene expression in non-cardiac cell pathways (Shah et al., 2021). *LMNA* mutations accelerate the degradation of SIRT1, leading to mitochondrial dysfunction and oxidative stress, which in turn activates the ROS–CAMLII–RYR2 pathway and induces arrhythmia (Qiu et al., 2024).

### 5.2 Hypertrophic cardiomyopathy

Hypertrophic cardiomyopathy (HCM) is a hereditary cardiomyopathy characterized by left ventricular hypertrophy (LVH) without secondary LVH caused by other diseases (Melas et al., 2023). This disease is also characterized by myocardial contractility, diastolic dysfunction, myofibrillar abnormalities, and fibrosis (Marian and Braunwald, 2017). Of the known causative genes, *MYH7* and myosin-binding protein C (*MYBPC3*) are the most common, accounting for approximately half of familial HCM cases (Kaski et al., 2009; Millat et al., 2010). Seeger et al. demonstrated that activation of the nonsense-mediated decay (NMD) pathway, a major pathogenic mechanism in HCM, was shown by the study of *MYBPC3* premature termination codon mutations (Seeger et al., 2019). Wu et al. proposed the dysregulation of Ca^2+^ cycling and elevation of intracellular Ca^2+^ as central mechanisms underlying arrhythmic phenotypes in the pathogenesis of HCM (Wu et al., 2019). Ewoldt et al. demonstrated increased collagen deposition and tissue stiffness were observed in cardiac microtissues derived from *MYH7* mutant hiPSC-CMs, resulting in impaired contractility (Ewoldt et al., 2024). They also demonstrated that paracrine signals secreted by HCM-mutant hiPSC-CMs activated stromal cells and that inhibiting epidermal growth factor (EGF) signaling suppressed stromal cell proliferation and ECM remodeling.

In trials using existing drugs, the Ca^2+^ channel inhibitor, verapamil, has been reported to substantially improve HCM phenotypes, including myocardial hypertrophy, Ca^2+^ handling abnormalities, and arrhythmias (Lan et al., 2013; Wu et al., 2019). *MYH7* mutant hiPSC-CMs generated on the Biowire platform exhibited chronic mavacamten treatment effects, including shortened relaxation time, reduced APD_90_ prolongation, increased expression of CICR-related genes, decreased mRNA and protein expression levels of BNP, and increased sarcomere length and reduced sarcomere disarray, thereby preventing many pathological features (Wang et al., 2023a).

### 5.3 Channelopathies

Long QT syndrome (LQTS) is a hereditary disorder characterized by prolonged ventricular repolarization and increased risk of Torsade de Pointes-type ventricular arrhythmias. This type of arrhythmia can cause arrhythmic syncope or sudden cardiac arrest (Moss et al., 1991). Of the numerous cases of LQTS reported to date, LQT1, caused by mutations in the *KCNQ1* gene; LQT2, caused by mutations in the *KCNH2* gene; and LQT3, caused by mutations in the *SCN5A* gene, account for approximately 90% of all cases (Schwartz et al., 2012). Moretti et al. were the first to use patient-derived hiPSC-CMs for modeling LQT1 (Moretti et al., 2010). The action potential duration (APD) of hiPSC-CMs derived from patients with LQT1 (LQT1–hiPSC-CMs) was substantially prolonged compared to that of control hiPSC-CMs. LQT1–hiPSC-CMs exhibited a 70%–80% reduction in *I*_Ks_ currents and high susceptibility to isoproterenol-induced tachyarrhythmias, which were reversed by beta-blockers. Takaki et al. demonstrated that the LQT1–hiPSC-CM model accurately reproduced LQT1 phenotypes, including abnormal channel activity and increased arrhythmogenicity (Takaki et al., 2019).

Matsa et al. generated hiPSC-CMs derived from patients with LQT2 (LQT2–hiPSC-CMs), and compared the sensitivity of control hiPSC-CMs and LQT2–hiPSC-CMs to agonists or antagonists of β-adrenergic receptors and potassium channels (Matsa et al., 2011). The LQT2–iPSC-CMs exhibited prolonged APD and increased sensitivity to early after-depolarization (EAD) following isoprenaline treatment compared to control hiPSC-CMs. Mesquita et al. used LQT2–hiPSC-CMs and CRISPR/Cas9-edited healthy hiPSC-CMs with the R534C mutation and demonstrated that both cell lines exhibited prolonged APD and reduced *I*_Kr_, corresponding to the clinical phenotype of patients with LQT2 (Mesquita et al., 2019). Chang et al. generated LQT2-hiPSC-CM models using CRISPR/Cas9 and demonstrated that these models exhibited QT prolongation, arrhythmia, and sensitivity to other ion channel inhibitors (Chang et al., 2021).

LQT3 patient-specific hiPSC-CMs (LQT3–hiPSC-CMs) had a prolonged APD compared to control hiPSC-CMs (Terrenoire et al., 2012; Ma et al., 2013). Mexiletine reversed the elevated late Na^+^ channel current and prolonged APD in LQT3–hiPSC-CMs. Structural analogs of mexiletine with greater potency and selectivity for I_NaL_ decreased APD prolongation and suppressed EADs were identified using large-scale functional screening of LQT3–hiPSC-CMs (McKeithan et al., 2020).

BrS is caused by mutations in the *SCN5A* gene. Mutations in *SCN10A*, which encodes voltage-gated sodium channels and *CACNA1C*, which encodes L-type calcium channels, have also been identified (Begovic et al., 2024). The hiPSC-CMs derived from a patient with BrS (BrS–hiPSC-CMs) exhibited higher arrhythmia, slower depolarization, and irregular calcium signaling than control hiPSC-CMs (Sun et al., 2023). BrS–hiPSC-CMs with *SCN10A* gene mutations (c.3803G>A and c.3749G>A) recapitulated the single-cell phenotypic characteristics of BrS, including a substantially reduced peak sodium channel current and reduced ATX II-sensitive and A-887826-sensitive late sodium channel currents compared to the control hiPSC-CMs (El-Battrawy et al., 2019). The CACNA1C-p.N639T mutation was introduced into healthy hiPSC lines using CRISPR/Cas9 and differentiated the cells into hiPSC-CMs; the mutation caused prolonged action potentials and delayed voltage-dependent inactivation of the calcium channel _V_1.2 (Chavali et al., 2019).

SQT is typically associated with mutations in six genes that encode potassium and calcium channels (*KCNQ1*, *KCNH2*, *KCNJ2*, *CACNA1C*, *CACNB2*, and *CACNA2D1*). Of these gain-of-function mutations, *KCNH2* causes the most common subtype, SQT1 (Fernández-Falgueras et al., 2017). The first hiPSC-based model of SQT1 was reported by El-Battrawy et al., using hiPSC-CMs derived from a patient with SQT1 (SQT1–hiPSC-CMs) with the KCNH2 N588K mutation, which reproduced the single-cell phenotype of SQT (El-Battrawy et al., 2018). SQT1–hiPSC-CMs with a T618I missense mutation in the *KCNH2* gene exhibited abnormal AP phenotypes compared to the control and gene-corrected hiPSC-CMs.

In summary, hiPSC-CMs derived from patients with cardiac channelopathies or edited using CRISPR/Cas9 reproduced the *in vitro* electrophysiological characteristics of ion currents, action potentials, calcium transients, and proarrhythmic and arrhythmic events, providing physiologically-relevant disease models and drug-screening platforms.

### 5.4 Myocardial infarction/ischemic reperfusion injury

MI is a cardiovascular disease characterized by high incidence and mortality rate, arising from the progressive narrowing of the coronary arteries. Coronary artery occlusion disrupts the oxygen and nutrient supply to the myocardium, leading to the accumulation of waste products. This is followed by CM death, which triggers fibrosis, inflammation, and ventricular remodeling, ultimately resulting in heart failure (Liu et al., 2024b). Gaballah et al. evaluated the effects of hypoxic stress on hiPSC-CM functionality by exposing hiPSC-CMs to hypoxic conditions (1% O_2_, 5% CO_2_, and 94% N_2_). Hypoxia induced marked arrhythmia and reduced Ca^2+^ transient amplitude in hiPSC-CMs, whereas addition of the Ca^2+^ enhancer, levosimendan, ceased the arrhythmia (Gaballah et al., 2022). Damage to the heart tissue that occurs when blood flow is restored to the infarcted tissue is called IR injury (He et al., 2022). Recently, modeling of this IR injury has also been reported. Hidalgo et al. reported that hiPSC-CMs cultured in a maturation medium rich in fatty acids exhibited substantially increased sensitivity to oxygen concentration changes compared to hiPSC-CMs cultured in standard media, with an approximately 25% difference in cell death occurring after hypoxia (Hidalgo et al., 2018). AdCMs rely primarily on fatty acid β-oxidation, and this increased dependence on mitochondrial respiration is considered key to their susceptibility to hypoxia and reperfusion injury. Veldhuizen et al. developed a microfluidic device using hiPSC-CMs matured via patterning techniques and co-culture with cardiac fibroblasts. They observed no difference in cell death under hypoxic conditions between this and a monolayer culture platform after 24 h of exposure to a 1% O_2_ hypoxic environment. However, a marked increase in cell death was observed in their model after 1 h or 24 h of reperfusion, successfully modeling IR injury (Veldhuizen et al., 2022). Song et al. reproduced hypoxia-induced ischemia by culturing hiPSC-COs in glucose-depleted medium supplemented with 50 μM cobalt chloride (CoCl_2_) (Song et al., 2024). They reproduced ischemia–reperfusion (IR) by exposing cells to a high-glucose and high-calcium environment. This model reproduced acute MI characterized by cardiomyocyte death, functional impairment, collagen deposition, and impaired calcium ion handling. Ellis et al. created an IR injury model by culturing hiPSC-CMs and hiPSC-ECs on a HoC under anoxic conditions (0.1% O_2_) without flow for 3 h, followed by the reintroduction of flow (Ellis et al., 2022). Their MI-on-chip model showed similar increases in miR-1, miR-208b, and miR-499 levels compared to human plasma samples collected before and after IR.

In summary, the MI and IR injury models used diverse culture conditions and platforms, requiring caution when comparing their respective results. However, these models closely approximate disease phenotypes by mimicking the disease environment. Advanced model standardization and high-throughput capabilities have the potential to replace preclinical trials.

## 6 Drug-induced cardiotoxicity

Many drugs have been withdrawn from the market due to drug-induced cardiotoxicity, highlighting the need for robust cardiotoxicity testing models to reduce the risk of cardiotoxic drugs in human clinical trials (Destere et al., 2024). Current preclinical models are heavily reliant on animal models, which are costly and have low throughput. Given the tendency toward reduced animal model use, *in vitro* models are becoming increasingly essential. Unlike animal models, hiPSC-CMs exhibit no differences in gene expression and can be produced indefinitely, making them promising models for novel toxicity testing (Pognan et al., 2023). Altrocchi et al. developed a cardiotoxicity assessment model using a 48-well multi-electrode array (MEA) plate (Altrocchi et al., 2023). They used this model to evaluate acute and delayed drug-induced cardiotoxic effects of reference compounds on clinically-known cardiotoxic outcomes. The evaluated drugs included not only known classical cardiotoxic agents (doxorubicin and BMS-986094) but also low-cardiotoxicity agents (erlotinib) and high-cardiotoxicity tyrosine kinase inhibitors (sunitinib, vandetanib, and nilotinib). This assay could reproduce various cardiotoxicities, including prolonged field potentials, altered beating rates, arrhythmic events, and decreased impedance. Jang et al. generated COs from patients with breast cancer who developed doxorubicin-induced cardiotoxicity (DIC) and those who did not and evaluated the drug responses in individual patients (Jang et al., 2024). Compared to COs from patients with breast cancer who did not develop DIC, those from patients who developed DIC showed increased sensitivity to doxorubicin, reduced survival rates, elevated expression of apoptosis-related genes, and a more pronounced decrease in beating frequency. Liu et al. identified carbonic anhydrase 12 (CA12) as a promising drug target that could mitigate DIC by combining hiPSCs with CRISPR interference and activation screening (Liu et al., 2024a). Genetic inhibition and deletion of CA12 protected hiPSC-CMs from DIC-induced cell death, abnormal myogenic segmentation, impaired calcium signaling, and electrophysiological abnormalities. Furthermore, they identified indisulam as a CA12 antagonist using a molecular docking approach and demonstrated its ability to attenuate DIC in EHT and DIC mouse models.

As described above, various culture platforms are used to evaluate DIC (Table 1). However, DIC studies using hiPSC-CMs have rarely included maturation approaches, indicating that the hiPSC-CMs used in these studies could be considered immature. As discussed in Chapter 2, immature hiPSCs and AdCMs differ widely in morphology, electrophysiology, and metabolism. Therefore, not all drugs exhibit the same behavior in both cell types. Consequently, unless cardiotoxicity is evaluated at the developmental stage, it is preferable to enhance the maturity of hiPSC-CMs before DIC assessment.

## 7 Limitations for clinical translation

The primary limitation in producing disease models using hiPSC-CMs is their immaturity compared with AdCMs. As described in Chapter 4, various maturation approaches have been developed; however, even when combined, they do not achieve the same level of maturity as AdCMs (Ronaldson-Bouchard et al., 2018; Li et al., 2025). Maturation of these hiPSC-CMs is particularly important for producing models of diseases such as *TTN*tv-DCM, which typically occurs in older patients, and diabetic cardiomyopathy (DbCM), which is characterized by hyperglycemia and insulin resistance. The *TTN*tv-DCM models are generally produced using EHT. Although these models reduced contractility and appeared as a DCM phenotype, arrhythmia phenotypes did not emerge (Roberts et al., 2015; Fomin et al., 2021; Huang et al., 2023). In DbCM models, immature hiPSC-CMs may typically show resistance to the harmful effects of hyperglycemia because they utilize glucose metabolism (Bowman et al., 2019; Purnama et al., 2022).

Another limitation of these studies is the lack of established, standardized methods for differentiating, maturing, and producing disease models using hiPSC-CMs. This hinders their application in preclinical models. Disease and toxicity testing models reported to date have utilized hiPSC-CMs produced using different media and differentiation induction methods. Differences were also observed in the combinations of hiPSC-CM maturation approaches and culture platforms used for disease model production. For example, differences in the culture medium used for maintaining hiPSCs can lead to variations in the subtypes of cardiomyocytes produced (Nakashima and Tsukahara, 2025). The accumulation of small differences in each of these factors could potentially cause large variations between the models. For large-scale preclinical studies requiring robust validation and reproducibility, it is necessary to establish standardized models to avoid such variability. However, once these two limitations are resolved, the model will be superior to animal and cell models traditionally used in preclinical testing.

## 8 Conclusion

Due to the limited availability of human heart tissue, there is a need for an easily-obtainable and scalable human-derived cell/tissue system that expresses structural and functional characteristics similar to those of native CMs and carries patient genetic information. The hiPSC-CMs have the potential to meet these requirements and serve as powerful tools for disease modeling and drug discovery. They can be used to elucidate disease mechanisms and predict the effects of candidate compounds by focusing on the electrophysiology, cellular signaling, metabolism, and contraction mechanisms of CMs. However, it is important to recognize several differences between AdCMs and hiPSC-CMs in studies that target diseases that develop in adults. These differences include variations in ion channel dynamics, contractile function, structural differences, sarcomere isoforms, and metabolic function. This review summarizes the main characteristics of hiPSC-CM maturation, known regulatory factors of this process, and disease models produced using these factors. Future efforts to enhance the maturity of hiPSC-CMs and develop high-throughput, reproducible, and three-dimensional culture platforms will lead to improved robustness and accuracy, thereby contributing to advancements in disease modeling, drug efficacy, toxicity testing, and mechanistic studies.
